# A new quantitative 3D approach to imaging of structural joint disease

**DOI:** 10.1038/s41598-018-27486-y

**Published:** 2018-06-18

**Authors:** T. D. Turmezei, G. M. Treece, A. H. Gee, R. Houlden, K. E. S. Poole

**Affiliations:** 1 Cambridge University Engineering Department, Cambridge, UK; 2 Department of Radiology, Norfolk and Norwich University Hospital, Norwich, UK; 3 Department of Medicine, University of Cambridge, Cambridge, UK

## Abstract

Imaging of joints with 2D radiography has not been able to detect therapeutic success in research trials while 3D imaging, used regularly in the clinic, has not been approved for this purpose. We present a new 3D approach to this challenge called joint space mapping (JSM) that measures joint space width in 3D from standard clinical computed tomography (CT) data, demonstrating its analysis steps, technical validation, and reproducibility. Using high resolution peripheral quantitative CT as gold standard, we show a marginal over-estimation in accuracy of +0.13 mm and precision of ±0.32 mm. Inter-operator reproducibility bias was near-zero at −0.03 mm with limits of agreement ±0.29 mm and a root mean square coefficient of variation 7.5%. In a technical advance, we present results from across the hip joint in 3D with optimum validation and reproducibility metrics shown at inner joint regions. We also show JSM versatility using different imaging data sets and discuss potential applications. This 3D mapping approach provides information with greater sensitivity than reported for current radiographic methods that could result in improved patient stratification and treatment monitoring.

## Introduction

Joint diseases such as osteoarthritis, inflammatory arthritis, and gout are estimated to affect 25% of the US population, making them a leading cause of morbidity and poor quality of life from painful loss of function^1^. These diseases will become an increasingly important clinical challenge as populations grow and become proportionally older. Imaging plays a key role alongside clinical review of patients with joint disease because it can depict individual joint involvement while avoiding the need for invasive tissue sampling^2^. It can also be used repeatedly and safely within recognised limits, which is important for follow-up of these chronic conditions. Yet our ability to detect structural changes that can identify disease early, monitor progression, and predict treatment response has been frustratingly limited.

Radiography, magnetic resonance imaging (MRI), and ultrasound are the accepted standards for imaging assessment of joint disease and have been the preferred modalities for joint imaging in clinical research trials. The use of radiography is near universal (see below), while MRI has commonly been performed in assessment of cartilage, e.g. looking for thinning in osteoarthritis^3^, and ultrasound in assessment of joint inflammation, e.g. in rheumatoid arthritis^4^. However, the US Food and Drug Administration (FDA) has to date only approved 2D radiographic joint space assessment for clinical trial imaging endpoints^5,6^.

For osteoarthritis, the most common joint disease, this endpoint is defined as progression in 2D radiographic joint space narrowing that requires manual measurement of joint space width and a new definition of progression for each study. This is also the OARSI-OMERACT recommendation for setting osteoarthritis clinical trial endpoints^7,8^. Subjective semi-quantitative Kellgren and Lawrence (K&L) radiographic grading has also been widely used, but this has been shown to suffer from multiple varied definitions and problematic reliability^9,10^. Other similar radiographic scores have been applied in the assessment of rheumatoid arthritis^11^, but these have failed to demonstrate structural damage adequately because of limitations in analysis methods^6^.

Therapeutic trials have been described as being *“in desperate need”* of reliable biomarkers for optimising the chance of successful outcomes. While there has been some promise from compositional MRI techniques, links between disease and parameters such as T2, T1rho, ultrashort echo and dGEMRIC times are yet to be clearly established^12,13^. No single imaging biomarker has proved outstanding.

These modalities continue to be instrumental in the assessment of joint disease, yet there has also been growing support for the use of computed tomography (CT) in this role, mainly because of its strength in visualisation of mineralised joint tissues, rapid acquisition time, and 3D reconstruction capabilities^14,15^. This may in part be from recognising the value that 3D visualisation of bone has in understanding joint morphology and pre-operative planning^16,17^.

If we take 2D radiographic joint space narrowing as the recognised surrogate marker for structural disease progression, it seems logical, although not foregone, that a 3D technique may yield information more representative of joint phenotype and disease status; CT works well in 3D because of its ability to reconstruct data volumes from helical data acquisitions. In this paper we consider a new 3D approach to analysis of CT imaging data in terms of identifying structural joint space changes. Before we do this, however, it is important to understand what technical challenges need to be overcome for success with a 3D imaging strategy.

Making accurate measurements in any imaging data requires understanding of the limits to image resolution. Imaging system blur, which sets this limit, is usually modelled as a point spread function (PSF) with a Gaussian profile. For CT, multiple factors such as detector size, physical properties of the system, and image reconstruction techniques all contribute to this blur, reducing capability to depict structures accurately. Put simply, features of a thickness below the image resolution limit are blurred and cannot be measured accurately. Modern CT imaging systems can be adapted to optimise image resolution. However, substantial increases in spatial resolution are only achieved at the cost of increased noise unless radiation dose is also increased, which is undesirable.

Unfortunately, joint space widths are frequently below this feature size, hence the need to remove image system blur for accurate measurement. This can be achieved with a constrained deconvolution approach, where the constraint (in this case prior knowledge of bone density around the joint space) acts to limit noise in measurement while still improving accuracy. In order to work in a 3D framework, this process must be repeated at multiple locations and in multiple directions. Furthermore, this technique requires validation so that it can be trusted.

3D joint space width measurement using CT technology has precedents: two techniques have been described in the context of rheumatoid arthritis using high resolution peripheral quantitative CT (HR-pQCT)^18,19^. However these techniques were restricted to small joints of the hand and cannot be transferred to larger joints such as the knee or hip because of their limited field of view (FOV) and radiation dose restrictions. Furthermore, quantified results were not presented in 3D, nor were they validated against a gold standard with higher spatial resolution to establish their accuracy and precision.

There are known limitations to operator-dependent interpretation of radiographs and manual measurement of joint space width. Therefore we propose a new quantitative 3D approach to evaluating joints as an alternative. We present a new semi-automatic image analysis technique called joint space mapping (JSM) that uses routine clinical CT imaging data to deliver 3D joint space width maps of human joints. We propose the utility of CT data, analysed via JSM, in predicting disease status by first demonstrating technical validation and interoperator reproducibility at the hip joint, then versatility across several joints. This new approach also forms the basis for wider applications that we discuss.

## Results

### 3D joint space patch creation

**Figure 1:**
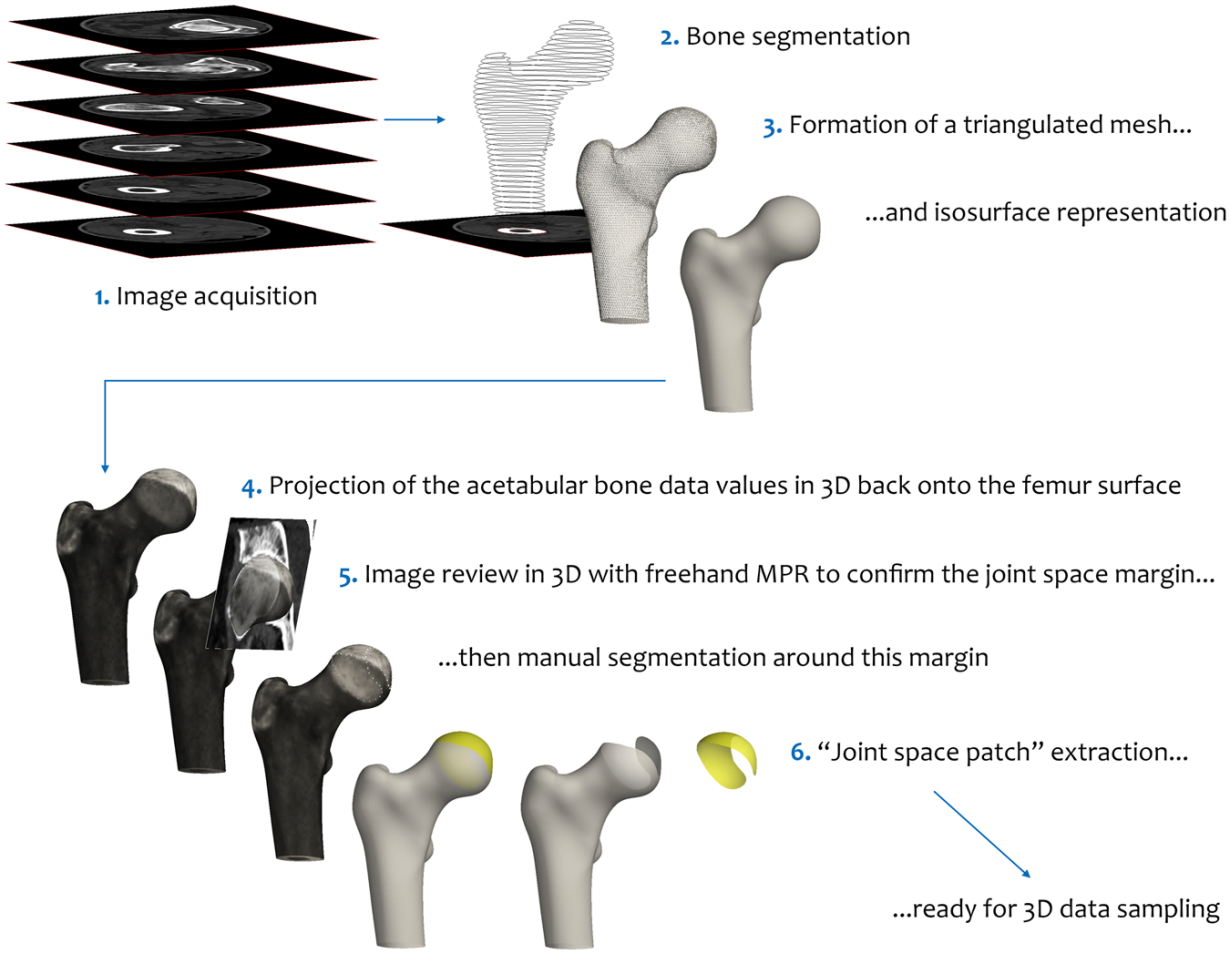
Joint space patch. Creation of the “joint space patch” from clinical CT imaging using Stradwin software. We perform manual segmentation of the proximal femur (step 2) taking ~5 minutes per femur, but this can be supplemented by automatic segmentation. This leaves segmentation of the joint space patch along the joint space margin on the femoral surface as the only other manual step, taking ~2 minutes per femur. Fig. S1 shows a magnified view of step 5.

Assessment of joint space width in 3D requires a framework on which to perform each deconvolution-based measurement. Figure 1 shows the steps we take to deliver this framework, called the “joint space patch”, summarised here:

1. Acquisition of axial clinical CT imaging data, in this case with 1.5 mm reconstructed slice thickness, 0.31 × 0.31 mm pixel spacing, and a smooth post-processing reconstruction kernel, all standard clinical acquisition parameters;
2. In-frame bone segmentation: we perform this manually (~5 minutes per femur), but this could alternatively be performed with an automatic segmentation technique^20,21^;
3. Automatic formation of a triangulated mesh and surface representation of the proximal femur^22^;
4. Automatic projection of acetabular bone onto the proximal femoral surface using the 3D imaging data volume;
5. Femoral surface review with freehand multiplanar reformatting (MPR) guiding manual segmentation of the joint space margin on the femoral surface (~2 minutes per femur); then
6. Automatic extraction of the joint space patch along this boundary from the surface of the femur for subsequent data sampling in 3D.

Steps 4 and 5 are the novel processing stages that are unique to the JSM algorithm. In step 4, the highest CT data value (in Hounsfield units, HU) sampled through the 3D data volume within ~5 mm along a normal to each vertex in the proximal femoral mesh is projected back onto the proximal femur surface. This means a bright patch appears on the femur where there is opposing acetabular bone, and a dark patch where there is only soft tissue. The edge of this bright patch is confirmed as the margin of the joint space by reviewing the relationship between the femur and the acetabulum with a 3D MPR tool in step 5, followed by manual segmentation of the joint space boundary on the proximal femoral surface (Figs 1 and S1).

All post-processing steps (2–6) are performed in Stradwin, software developed in house that is freely available to download and use (http://mi.eng.cam.ac.uk/~rwp/stradwin/). Full details of CT image acquisition and analysis steps are in the Materials and Methods section.

### 3D joint space data sampling

The extracted joint space patch is now ready to use as the framework for the next steps, noting that this process does not rely on any prior segmentation of the acetabular or femoral articular surfaces. First we estimate the peak cortical density of bone in HU from the image volume across the original proximal femoral mesh and assume that this is a fixed peak value across the joint space patch, as per the technique implemented by Treece *et al*.^23^. We then sample again through the imaging data volume, but this time only at the vertices in the joint space patch mesh. Each 1D sample line arising perpendicular to a vertex results in an interpolated linear density profile. We assume that the image system blur along the line can be modelled as Gaussian in shape, though the extent of this blur is allowed to vary at each vertex. Surrounding bone is modelled as a double-peak^24^, each peak representing an opposing bone surface at the joint space (hence initial femur segmentation is only a guide). An optimiser fits a blurred model to the interpolated line data at each vertex, then blur is removed in the deconvolution step using a fixed peak density constraint to determine bone edges as step functions in the line data. The distance between the outer bone edges is delivered as the joint space width. Importantly, this process only requires the original femur segmentation to be within ±2 mm of the proximal femoral surface^25^, meaning that small variations, whether from manual or automatic segmentation, are well tolerated and have a negligible effect on resultant joint space width values. The difference between the original joint space patch extracted from the femoral surface and the JSM output femoral and acetabular surfaces is show in Fig. S2.

**Figure 2:**
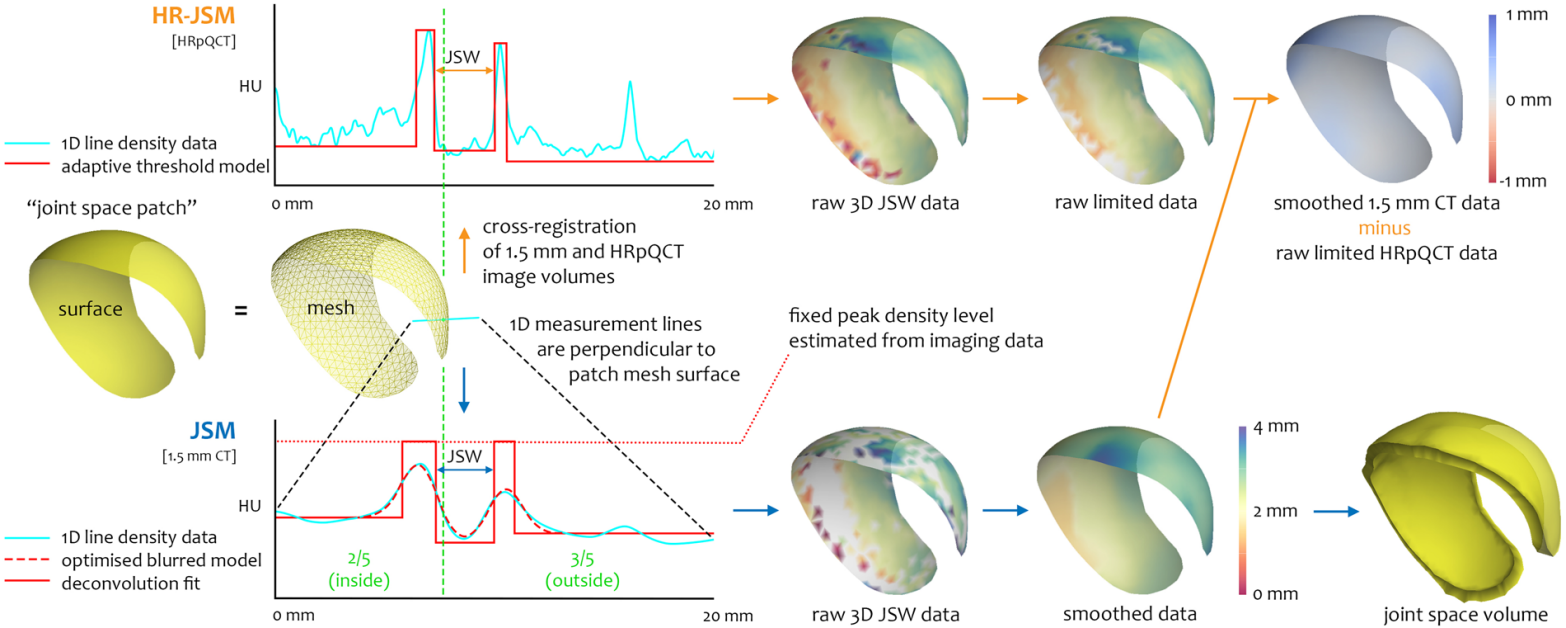
3D data sampling across the joint space. JSM (blue) and HR-JSM (orange) pathways for 3D joint space width measurement and validation. Joint space volume is delimited by the femoral and acetabular bone surfaces. While the HR-JSM pathway requires cross-registration of the clinical CT and HRpQCT image volumes and manual outlier removal, these are only required for the validation study. All steps in the JSM pathway are fully automated.

After automatic outlier removal and data smoothing, 3D joint space width is mapped across the joint space patch in colour, a process that can also produce a 3D joint space object. These steps are all automatic and shown in Fig. 2 as the JSM (blue) pathway, described fully in the Materials and Methods section.

### JSM technical validation study setup

A main aim of this study was to demonstrate that JSM can deliver accurate and precise measurements of joint space width in 3D. In order to achieve this, we validated joint space width values from clinical CT against high resolution peripheral QCT (HRpQCT) of the same cadaveric hip specimens. Since the use of cadaveric tissue removed restrictions on ionising radiation, we could use an HRpQCT image system with a PSF at least 10 times better than the clinical CT system, allowing a simpler adaptive threshold approach to measure the distance between outer bone surfaces as joint space width in the HRpQCT data^26^. This process is shown in Fig. 2 as the HR-JSM (orange) pathway.

Each dissected cadaveric hip was scanned in a custom-designed acrylic holder to maintain fixed positioning between clinical CT and HRpQCT acquisitions. Clinical CT was performed as above, described in further detail in the Materials and Methods section. HRpQCT voxels were reconstructed isotropically at 0.082 mm with a proprietary post-processing bone algorithm (Scanco Medical AG, Brüttisellen, Switzerland). We then cross-registered clinical CT and HRpQCT image volumes using mutual information similarity intensity-based registration so that the joint space patch from the clinical CT data was correctly aligned in the HRpQCT image volume from the same hip^27^.

Joint space width was then measured automatically across the patch with the HR-JSM adaptive threshold technique. Technical failures causing abnormally low or misplaced values from noise in the HRpQCT imaging data were removed using an in-house custom MATLAB GUI. Clinical CT and HRpQCT values were subtracted from each other across the ~65,000 matching vertices out of ~74,000 total over the 20 joint space patches to give accuracy and precision metrics. Here we use the term accuracy to represent mean bias as the mean of the absolute difference between the JSM measure and HRpQCT gold standard, and precision to represent the standard deviation of the difference between these two measures (i.e. the standard deviation of the bias).

We registered each acetabular surface to an average acetabular surface, allowing us to compare data from different subjects on the same framework (NB this is not the same surface as the original joint space patch cut from the proximal femur). Results from across all specimens could then be presented on this average surface in 3D. Full details of the clinical CT and HRpQCT acquisition, image set cross-registration, registration to the average surface, and validation steps are given in the Material and Methods section.

### JSM technical validation study results

**Figure 3:**
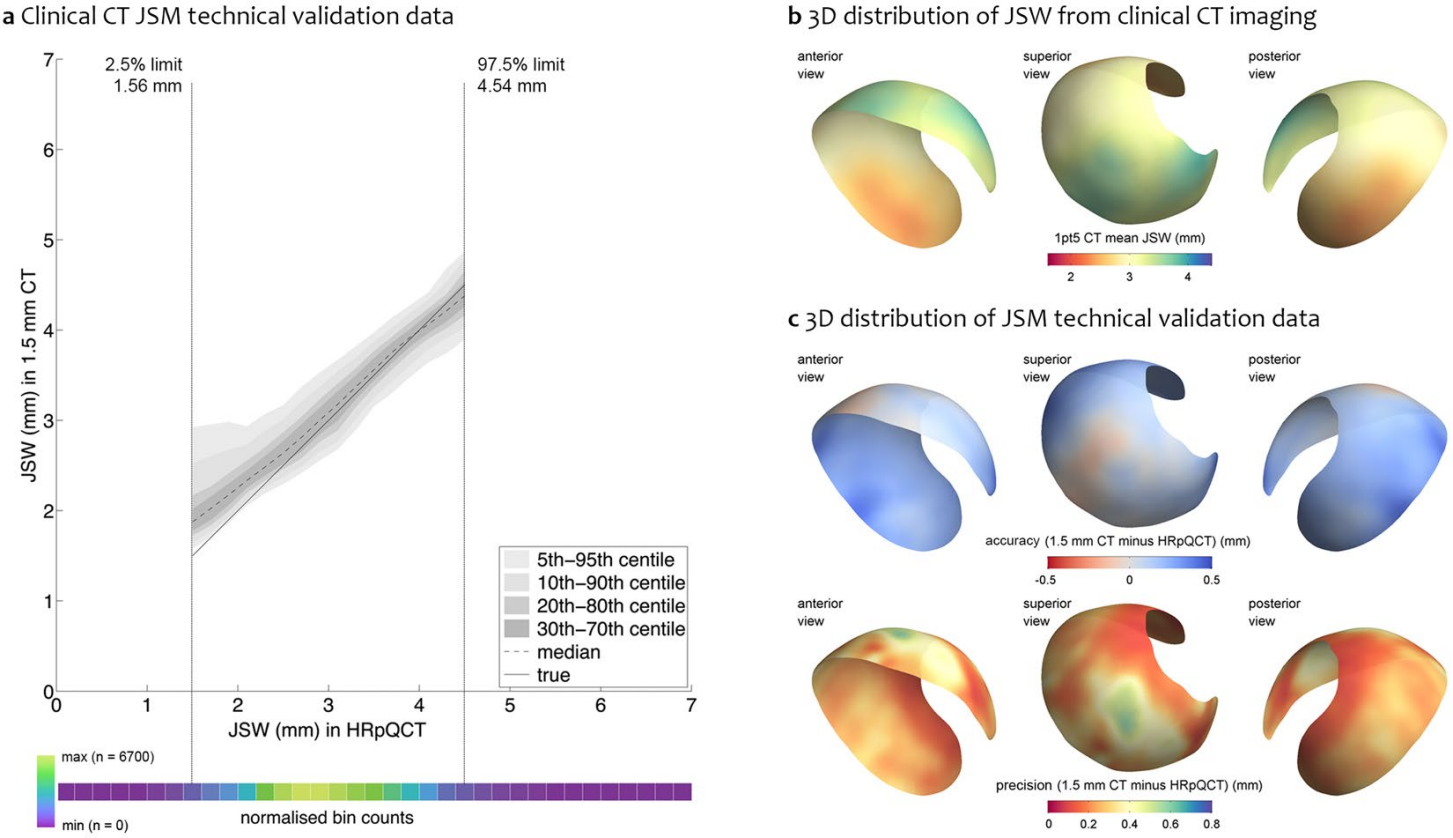
Technical validation study results. (**a**) Clinical CT *vs* HRpQCT validation data presented in 0.2 mm HRpQCT bins with median and centile values. The “true” line represents the target of equal 1.5 mm CT and HRpQCT measurement (i.e. no measurement bias). Outside the 95% data limits, the bin counts drop rapidly towards zero; (**b)** 3D distribution of average joint space width across the validation study group mapped onto the average acetabular surface; (**c**) 3D distribution of vertex-by-vertex validation data mapped onto the average surface as accuracy (clinical CT minus HRpQCT, top row) and precision (bottom row).

Global accuracy of JSM (mean bias across all joint space measurements) was +0.13 mm, a slight overestimation compared to the high resolution technique. Global precision (standard deviation of the bias across all joint space measurements) was ±0.32 mm. Figure 3a presents this data as clinical CT *versus* HRpQCT data, showing a slight overestimation bias at lower joint space width values. Figure S3 shows the full HRpQCT validation data in a scatterplot. Breakdown to individual validation plots (not shown) revealed that a few hip joints with lower joint space width ranges were the cause of this bias. Figure 3b shows mean clinical CT joint space width values from all samples on the average surface, revealing posterior joint space as narrowest at ~2.5 mm compared to a ceiling value of ~4 mm at the superior and anterior joint regions.

Figure 3c shows the vertex-by-vertex 3D distribution of accuracy and precision on the average surface, revealing slight underestimation to −0.2 mm at the superior joint space and more generalised overestimation to +0.2 mm in the anterior and posterior joint space. Largest bias was seen at the outer edges where values reach +0.5 mm, with precision ranging from 0.2–0.5 mm around the joint.

### JSM reproducibility study

Another aim of this study was to show that novel components of the JSM algorithm were reliable between operators of the manual steps. In order to do this we compared the output of two operators (T.D.T., creator of the technique, and R.H., a graduate student trained in the technique) performing blinded analysis of 30 hip joints with JSM. Proximal femurs in these data sets had already been manually segmented for a separate study, meaning that segmentation technique was not a variable factor^28^. All 30 hips were female with no previously known hip disease, but a mixture of side, severity of degenerative joint disease, and clinical CT acquisition parameters. This heterogeneity was intended to test interoperator reproducibility maximally. The acetabular surface output from each interaction was registered to the average surface so that results could also be presented in 3D. Full details of these steps are in the Materials and Methods section.

**Figure 4:**
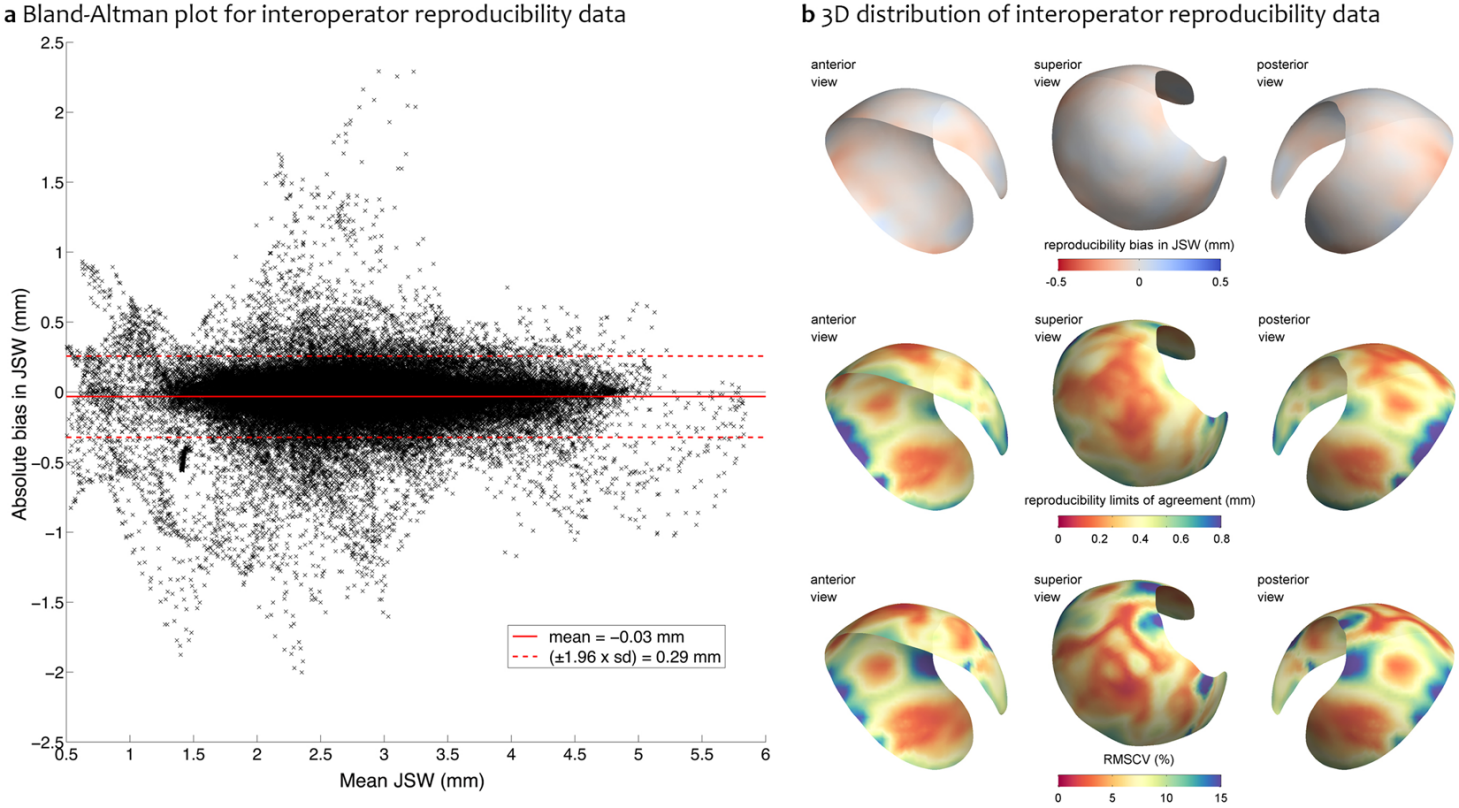
Reproducibility study results. (**a**) Bland-Altman interoperator reproducibility plot as operator 1 (R.H.) minus operator 2 (T.D.T) showing the mean reproducibility bias (solid red line) and limits of agreement (1.96 × SD of the reproducibility bias, dashed red lines); (**b**) 3D distribution of reproducibility bias, limits of agreement, and RMSCV mapped onto the average surface.

Global interobserver reproducibility bias calculated as the mean of the difference between the two operators was excellent at −0.03 mm, with limits of agreement ±0.40 mm (Bland-Altman plot, Fig. 4a), and a root mean square coefficient of variation (RMSCV) of 7.5%. Figure 4b presents the 3D distribution of these results mapped on the average surface, showing near uniform reproducibility bias around a zero value, with a few edge locations where the highest reproducibility bias values were ~0.2 mm. Limits of agreement were at best less than 0.2 mm in the central joint space, rising to 0.8 mm at some of the joint space margin; 95% of these values were less than 0.67 mm. RMSCV followed a very similar 3D pattern to limits of agreement, with lowest values in the central joint space around 2.5% with some inner regions increasing to 10%, reaching maximum values of 15–20% in only a few marginal joint space regions; 95% of RMSCV values were less than 12.9%. These worse interoperator reproducibility values are where we have already shown JSM to have least accuracy (Fig. 3c), likely from variability between users in joint space margin definition (step 5).

**Figure 5:**
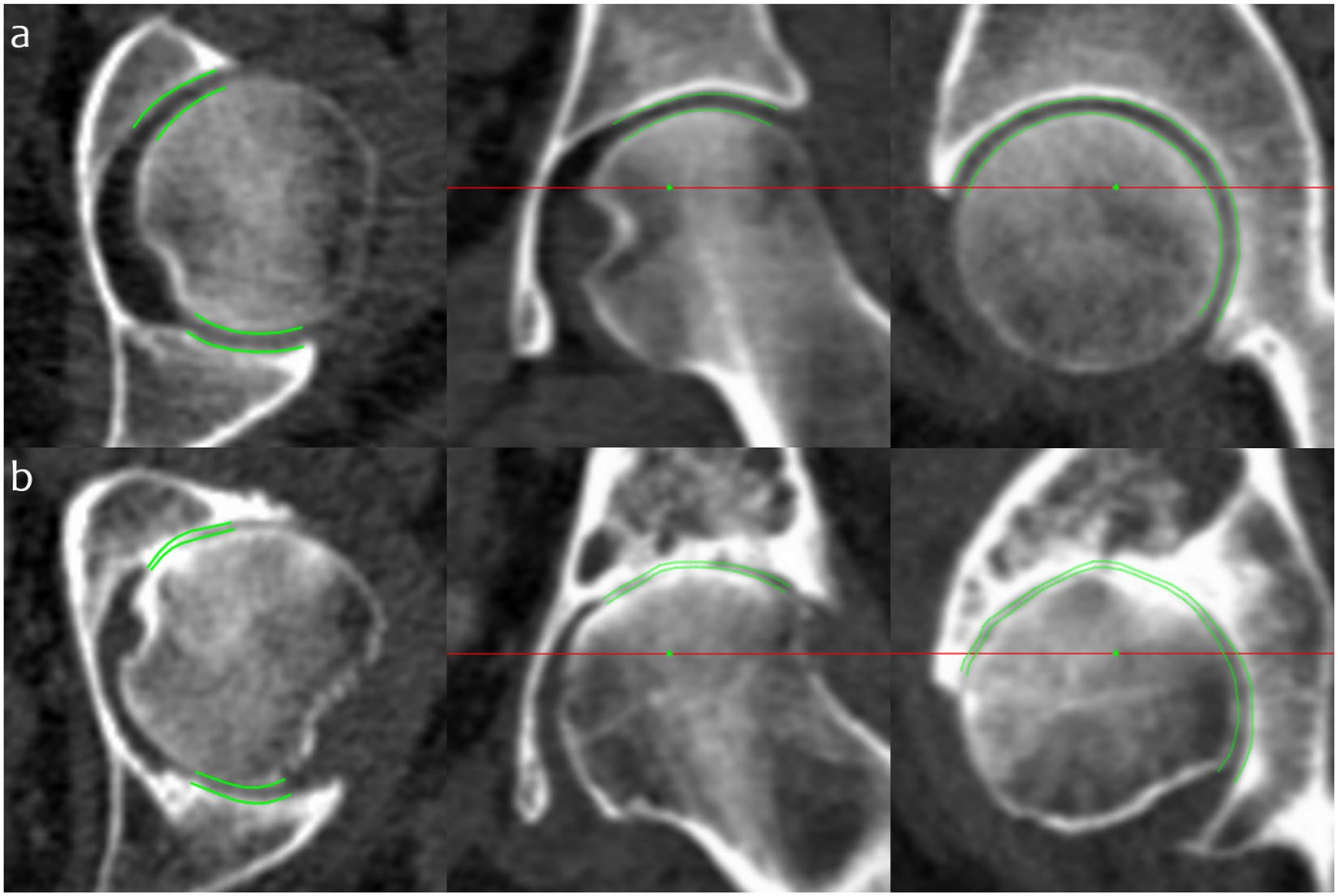
JSM performance at the extremes of disease. Joint space margins are shown in green as seen in axial, coronal and sagittal planes (left to right) at two left hips. (**a**) An individual with a radiographic K&L grade of 0 (no disease) and minimum joint space width of 2.0 mm. (**b**) An individual with a radiographic K&L grade of 3 (moderate disease) and minimum joint space width of 0 mm. This demonstrates that JSM is still able to represent joint space width as it approaches zero, important for analysis in advanced disease.

Heterogeneity in the reproducibility cohort also allowed us to show performance of JSM in extremes of radiographic disease. Figure 5 emphasises JSM robustness by showing output in one individual with a radiographic K&L grade of 0 (no disease) and minimum 2D joint space width of 2.0 mm, the other with a radiographic K&L grade of 3 (moderate disease) and minimum 2D joint space width of 0 mm.

## Discussion

In this paper we have presented a new semi-automatic image analysis technique called joint space mapping (JSM) that measures joint space width in 3D from routine clinical CT imaging data. We have shown it to be accurate and precise when validated against HRpQCT imaging, and robust in performance for different disease severities, scanning conditions, and joint locations.

The systematic but slight overestimation of joint space width (globally +0.13 mm) compared to the gold standard appeared to be skewed by a few hips with lower joint space width values. We believe this effect was from a generalised lower cortical bone density at the joint space than estimated across the whole proximal femur, and found that automatic bone density estimation at the joint space was too low in this small number of subjects. However we opted to accept this small overestimation bias for automation of this step, also meaning that individuals with low bone density (e.g. osteoporosis) would not be excluded from successful joint space mapping. Despite this, our accuracy is still below the in-plane pixel spacing of 0.31 mm, and substantially below the out-of-plane slice thickness of 1.5 mm.

We recognise that error in the validation process is compounded from the JSM, HR-JSM and the image cross-registration steps. Bearing this is mind, our validation results show a global precision of ±0.32 mm, with best precision values around 0.2 mm in the inner joint space regions, which can be considered excellent precision for this multi-step process.

We have demonstrated an inter-operator reproducibility bias of near zero (−0.03 m), with best limits of agreement (also known as smallest detectable difference) achieved to less than 0.2 mm at the inner joint space regions, which are likely to be the most important in terms of detecting disease-relevant joint space narrowing. These values of less than 0.2 mm are superior to the reported best of 0.45 mm for 2D radiographic joint space width assessment at the hip and 0.2 mm reported at the knee^7,29^. RMSCV values followed the same pattern, with best results of ~2.5% in the central joint space, and 95% of values less than 12.9%.

Although the validation range is presented as 1.56 to 4.54 mm, we were simply limited by the range of joint space widths in our validation samples. Any measurement problem solved by deconvolution has a lower threshold below which resolution cannot be achieved, but we are encouraged by successful performance of JSM in an individual with a minimum radiographic joint space width of zero (Fig. 5). There is also no technical reason why JSM shouldn’t perform as well at values above this range.

We also recognise the importance of consistent joint positioning when comparing joint space width values in 2D or 3D. A radiographic study by Goker *et al*. (2005) showed that although changing from neutral positioning to 30° of flexion at the hip had a significant effect on radiographic joint space width (increasing by 0.15 mm), there was no statistically significant difference between neutral and 15° flexion^30^. This means that small variations in hip joint position may not have a significant effect. Tom *et al*. (2016) also recently showed that metacarpophalangeal joint positioning had little effect on mean joint space width when repositioned at 20° intervals, with RMSCV values less than 5%^31^.

Furthermore, although gold standard assessment of radiographic hip joint space width in clinical trials is performed in the standing position, several studies have shown no significant difference in radiographic joint space width values when supine compared to standing^32^. It will be an important next step to use JSM to investigate whether differences in positioning and load-bearing have any effect on joint space width in 3D. A straightforward solution for prospective studies would be to perform knee and ankle CT in a standing position (as used in clinical practice), and to standardise supine hip positioning by strapping feet together.

**Figure 6:**
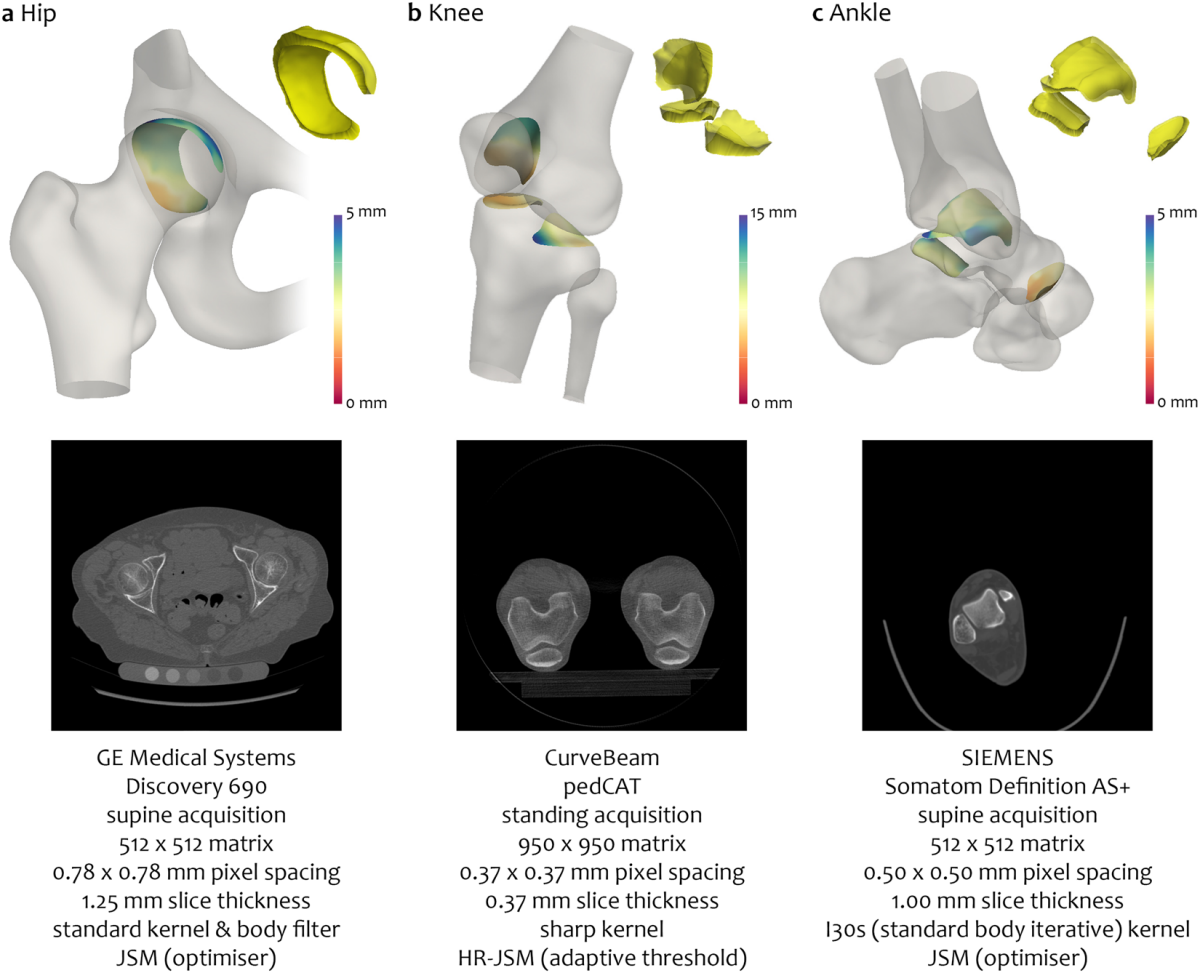
JSM at different joints. Feasibility of JSM application at various joints of the lower limb with different acquisition and reconstruction settings. Colour maps are displayed on the original patches *in situ* on the bone surfaces with joint space volumes inset in yellow.

We can also show proof-of-concept that JSM has versatility to be applied at different joints with a range of acquisition and reconstruction conditions (Fig. 6). When higher resolution 3D imaging is achievable at more peripheral joints, such as the knee, hands or feet, then an adaptive threshold technique (as for HR-JSM) could be used rather than a deconvolution approach (Fig. 6b).

Even though JSM sensitivity appears better than the reported current radiographic gold standard, using surface-based statistical analysis on JSM output could yield even greater sensitivity. Another important JSM output is the 3D joint surface on which joint space width values are displayed, subsequently allowing surface-based statistical analysis to be performed with statistical parametric mapping (SPM)^33^, the feasibility and success of which have already been demonstrated with cortical bone mapping at the hip^34,35^. An important benefit of SPM is the avoidance of region of interest (ROI) or sub-ROI averaging of values as a single test variable, as is usually performed with MRI parameters such as cartilage thickness and compositional data^3,36^. Using an average surface to analyse data with SPM also brings versatility to what can be tested because there are no restrictions on side (as data can be mirrored), follow-up of an individual, or comparing data across whole study groups. Sensitivity to change, i.e. what can be detected as significant against noise, is also a product of the SPM process. This means that the reproducibility bias and limits of agreement presented here would not be the whole picture because SPM can reveal systematic differences in data to a level well below the sensitivity limits of a measurement method.

Limitations on the use of CT from concerns over exposure to ionising radiation for patients and volunteers are being allayed by improved dose reduction techniques^37,38^ and a more widespread use of “very low” dose approaches^39^. These have opened the door for CT to be used in clinical and research settings. CT imaging has already been part of large cohort research studies such as the AGES-Reykjavik and Approach population studies (http://www.hjartarannsokn.is/index.aspx?GroupId=346; http://www.approachproject.eu), in which it has been used to assess different elements of musculoskeletal health. This means that research platforms already exist for exciting applications of JSM, which itself requires no special imaging conditions, just a routine clinical CT acquisition. Indeed, our first application study plans to describe the implementation and results of JSM in a subcohort of the AGES population with known hip disease outcomes, revealing relationships between 3D joint space width, hip pain, and future total hip replacement.

Given the use of CT in clinical assessment of joint diseases such as arthritis, femoro-acetabular impingement, avascular necrosis, and osteochondral injury, if its clinical utility can been established, JSM could have an important role in quantification and visualisation of joint space for clinical decision making. On a wider scale, it could be used to screen at-risk populations for disease progression, such as those with known arthritis, shape disorders that predispose to accelerated degenerative change, or elite athletes that undertake high stress-loading activity. We recognise that these ideas require further investigation, but this study has show that JSM has the ability to provide accurate and reliable information with the potential to influence decisions based on the progress of disease, therapeutic effects, or surgical intervention.

In conclusion, we have shown that JSM is an accurate, robust, and versatile technique that could be used to assess 3D joint space width distribution in individuals and across populations including but not limited to the hip, knee and ankle. Once its clinical utility has been established, this reliable CT-based 3D imaging analysis technique could represent an important step forward in quantitative analysis of joint disease as an alternative to 2D radiographic imaging, with applicability in research and clinical settings. When combined with 3D statistical analysis, there could also be potential to accelerate therapeutic development in clinical trials through improved patient stratification and disease characterisation.

## Materials and Methods

### JSM technical validation

#### Cadaveric sample preparation

The decision to use cadaveric human tissue for the technical validation study allowed us to ensure fixed positioning between clinical CT and high resolution pQCT (HRpQCT) imaging of the same joint specimens, which had to be dissected to fit in the HRpQCT scanner FOV. This decision also removed restrictions on ionising radiation use, allowing otherwise unattainable spatial resolution in HRpQCT imaging and, if necessary, repeat exposures. This validation study was approved by the University of Cambridge Council of the School of Biological Science Human Biology Research Ethics Committee on 21 May 2013 (HBREC 2013.09).

Hip joints were sourced from whole cadavers donated to the Human Anatomy Centre, Department of Physiology, Development and Neuroscience, University of Cambridge, U.K., from female donors who had given *ante mortem* consent for the use of their tissue in imaging research. All samples were obtained after whole-body embalming and dissected to include an intact right hip joint, with principal cuts across the superior acetabulum, lateral aspect of the pubic rami and proximal femoral shaft. Finer dissection keeping the hip joint intact was performed to fit specimens into a custom cylindrical acrylic holder (external diameter 115 mm to fit the HRpQCT FOV of 126 mm), allowing consistent positioning between scans and prolonged storage in 20% ethanol with no imaging artefact from the container.

The only exclusion criteria was the presence of any metalwork as identified by subsequent imaging; as such, no hips were excluded. Donor demographics at or just before death were mean ± SD age of 83 ± 14.8 years, height of 1.64 ± 0.09 m and weight of 54 ± 12.2 kg.

#### Validation imaging data acquisition

**Figure 7:**
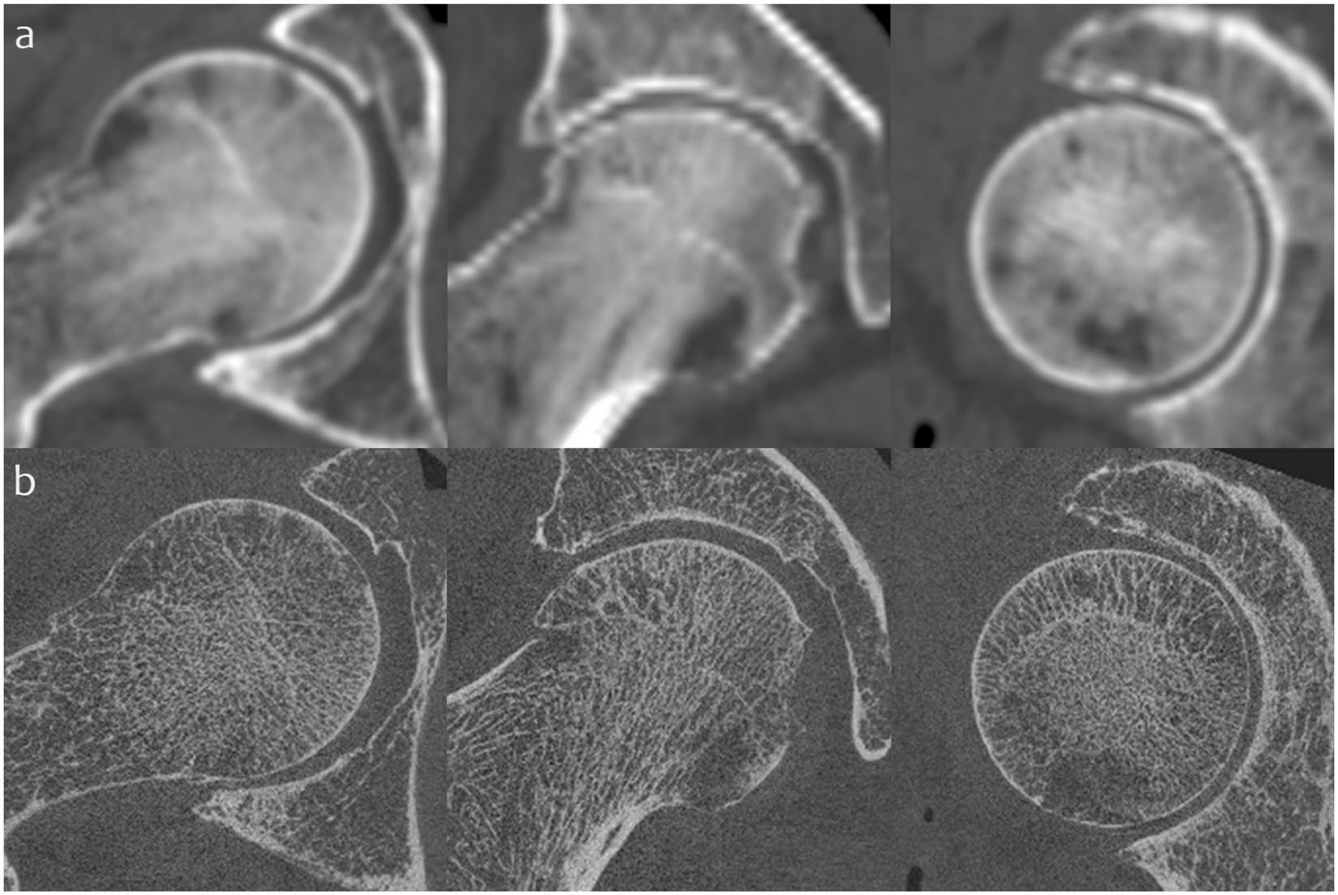
Validation study imaging acquisitions. (**a**) Clinical CT and (**b**) HRpQCT in axial, coronal and sagittal planes (left to right) for the same cadaveric right hip joint. Note out-of-plane (coronal and sagittal) stair-stepping artefact and generalised blur from the wider PSF in clinical CT compared to isotropic HRpQCT.

All clinical CT imaging was performed on a 64-slice Siemens Definition AS system (Siemens Medical Systems, Erlangen, Germany). The acquisition was helical with 0.8 mm pitch, 1.0 mm slice thickness, and a kVp of 80 kV. Reconstruction matrix was 512 × 512 with pixel spacing 0.31 × 0.31 mm (FOV 157 × 157 mm) and slice thickness 1.5 mm, all clinically standard and relevant parameters. Immediate post-processing at the imaging workstation was with the Siemens proprietary B20f smooth reconstruction kernel. All HRpQCT imaging was performed on a Scanco Xtreme CT system (Xtreme CT, Scanco Medical AG, Brüttisellen, Switzerland) with cone beam axial acquisition at a kVp of 60 kV. Reconstruction matrix was 1536 × 1536 with pixel spacing 0.082 × 0.082 mm (FOV 126 mm diameter) and slice thickness 0.082 mm giving isotropic voxels. A proprietary bone reconstruction post-processing kernel was automatically applied (Scanco Medical AG, Brüttisellen, Switzerland). Figure 7 shows comparison of these two acquisitions for the same hip.

#### Joint space patch creation

All post-processing steps were implemented in Stradwin (http://mi.eng.cam.ac.uk/~rwp/stradwin/), freely available in-house software developed by the Medical Imaging Group, Cambridge University Engineering Department, U.K. Initial semi-automatic manual in-frame segmentation of the proximal femur was performed in each slice of the clinical CT imaging (<5 minutes per femur). This does not need to be precise to the bone cortex and can equally be supplemented by automatic segmentation^20,21^. Shape-based interpolation with regularised marching tetrahedra is used to construct a triangulated mesh object of the proximal femur^22^.

A single femur consists of many thousands of vertices, each a potential data sampling point. Any overlying bone within ~5 mm of the proximal femoral surface is automatically projected onto each mesh vertex along its normal (i.e. perpendicular to the mesh surface), revealing a bright patch of opposing bony acetabulum on the femoral head surface. The perimeter of this bright patch is the margin of the joint space. The “joint space patch” is then manually segmented along this boundary at the femoral head surface using freehand MPR review as a guide to deliver a curved 3D horseshoe mesh representation of the joint space at its femoral aspect (Fig. S1). Each patch is re-triangulated at a slightly higher resolution to avoid data undersampling, in this case to contain approximately 4,000 vertices. These steps are summarised in the Results section and Fig. 1.

#### 3D joint space data sampling

The principle of JSM is to use a model-based approach to de-blur clinical CT data and automatically measure joint space width to a precision beyond the constraints placed by the blur (PSF) of the imaging system. The JSM measurement algorithm uses a development of the cortical bone mapping algorithm originally described by Treece *et al*. for the sub-pixel accurate measurement of cortical bone thickness from clinical CT imaging, most recently published in 2015^23^. We apply this with a double-peak model as implemented by Whitmarsh *et al*. in 2017 for use of cortical bone mapping in the presence of metal implants^24^.

Initial estimation of peak cortical density is performed across the whole proximal femoral mesh through the data volume using an empirical model fit described in Treece 2015^23^. This yields a fixed peak density value that is the constraint in the deconvolution process (Fig. 2 dotted red line in the JSM linear profile). We also assume tissue in the joint space (i.e. hyaline cartilage) to have a fixed value of 35 HU, as estimated from a region of interest (ROI) manually drawn over cartilage in HRpQCT imaging of a disarticulated femoral head specimen. Imaging data is then sampled in 3D along a line perpendicular to each joint space patch mesh vertex and interpolated to deliver a 1D linear density profile from across the joint space. Assuming the clinical CT imaging system PSF as Gaussian, an optimiser fits a blurred model to the interpolated line data (Fig. 2 dashed red line in the JSM linear profile). Then model-based deconvolution is performed on the linear profile data using the fixed peak cortical bone and joint space density values to identify the edges of opposing cortical bone. Joint space width is measured as the distance between the two outer bone surfaces in the deconvolution fit step model (Fig. 2 solid red line in the JSM linear profile).

After sampling at each vertex across the joint space patch, automatic steep gradient outlier removal (i.e. flattening false peaks in the joint surface) and error-based data smoothing deliver femoral outer bone surface co-ordinates and joint space width values for each vertex in the original joint space patch. This allows creation of a mesh representation of both the femoral and acetabular joint surfaces that in turn define the joint space volume; the difference between the original joint space patch and the JSM output joint surfaces is demonstrated in Fig. S2. These steps are summarised in Fig. 2 as the JSM (blue) pathway. Excluding manual in-frame segmentation, this takes less than 5 minutes for a single hip (~2 minutes for manual patch segmentation, moments for JSM processing).

#### Clinical and HRpQCT image set cross-registration

Each HRpQCT image volume is cross-registered with its matching clinical CT image volume using mutual information similarity intensity based registration implemented in Matlab 2013a (© 1984–2013, The MathsWork, Inc.)^27^. It is important to note that the accuracy of the cross-registration step has no effect on JSM performance, since it is only used in the validation process. Any slight improvements attainable in cross-registration would only serve to reduce the effect of any error from this step in our validation results. The PSF of the HRpQCT imaging system is reported by the manufacturer as 0.089 mm (http://www.scanco.ch/en/systems-solutions/clinical-microct/xtremect.html), compared to an estimated PSF of 0.87 mm in-plane and 1.00 mm out-of-plane for the clinical CT imaging data^23^. This makes a simple adaptive-threshold technique suitably accurate for measurement of joint space width in HRpQCT data (called HR-JSM)^26^. The same 3D imaging data sampling strategy is applied by HR-JSM around the joint space patch, again with interpolation of data along the sample line.

#### Adaptive threshold measurement in HRpQCT data (HR-JSM)

For measurement of joint space width in the HRpQCT data (HR-JSM), the peak in the linear density profile closest to the original mesh is assumed to be the femoral joint surface. A half-maximum value of the peak density is used as threshold for the femoral outer surface, with a search initiated outwards along the data line to identify the next cortical peak above this threshold. The same half-maximum approach is then applied to this new peak density to define the acetabular outer surface. Positional outliers with respect to the femoral surface and extreme value outliers are manually removed using a custom MATLAB GUI written by T.D.T. (Matlab 2013a, © 1984–2013, The MathsWork, Inc.). Both positional outliers and extreme value outliers are judged by eye using the GUI, which allows the user to see these being removed by narrowing the limits of accepted values against visual review of retained data points in 3D on the mesh surface. This step removes technical failures from the HRpQCT data (~10%), where the HR-pQCT algorithm failed to identify one or both of the bone surfaces on account of low peak cortical density. This process is key in maintaining the quality of the gold standard data. Again the distance between the two outer bone surfaces is taken as the joint space width. These steps are summarised in Fig. 2 as the HR-JSM (orange) pathway. No smoothing is applied to the HRpQCT data, meaning that some vertices do not have a joint space width value if there is a measurement failure or outlier removal.

#### JSM validation metrics

Joint space width values from clinical CT and HRpQCT are now spatially co-registered on the same joint space patch for each individual. HRpQCT data are limited to a 95% range to remove noise from the extremes where there are a low number of data values, then subtracted from clinical CT data and smoothed to produce a 3D colour bias map for each individual (Fig. 2 top right, blue-to-red data). This process is repeated for all the cadaveric hips. Global accuracy is calculated as the mean of clinical CT minus HRpQCT for all joint space measurements where vertices have a joint space width value from both, approximately 65,000 out of 74,000 total possible matches (~90%, with the remaining ~10% of HRpQCT values having been removed as positional outliers or failures). Global precision is calculated as the standard deviation of these difference values across all joint space measurements.

**Figure 8:**
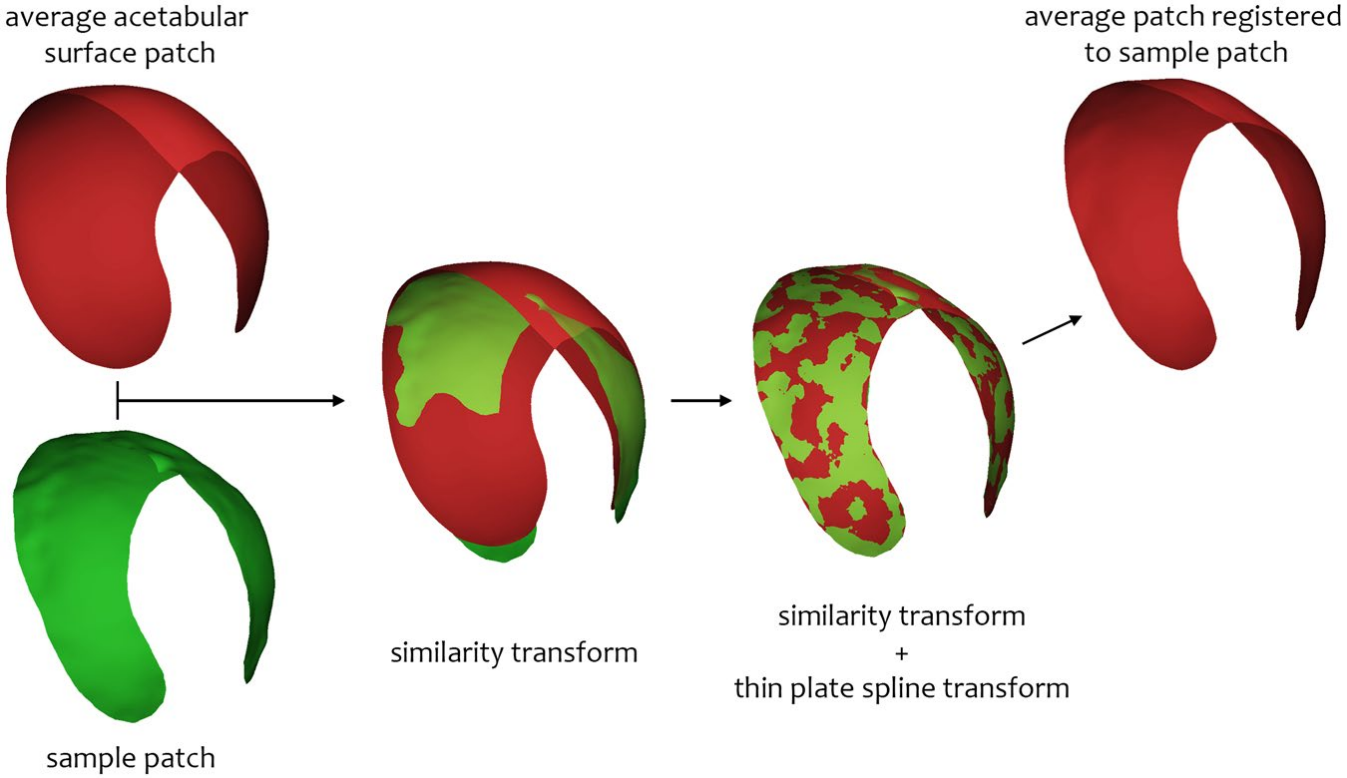
The process of registering the average acetabular surface to sample acetabular surfaces. Registration of the average patch (red) onto an individual sample patch produced by JSM (green) with similarity then thin plate spline transformations using the freely available in-house wxRegSurf software. This process enables data from each individual to be represented on the average surface after nearest-neighbour mapping from the sample to the average patch.

A surface- and edge-smoothed average of the 20 clinical CT acetabular surfaces produced by the JSM algorithm (i.e. not by any manual segmentation) is used as an average joint space representation with approximately 2,300 vertices. Note these are not the same object as the initial joint space patches cut from the femoral surfaces. Each sample acetabular surface is registered to the average surface using a similarity transformation then a thin plate spline transformation^40^ using freely available in-house software wxRegSurf (http://mi.eng.cam.ac.uk/~ahg/wxRegSurf/) (Fig. 8). Joint space width values are transferred from the acetabular surface onto the nearest vertex on the average surface then smoothed, meaning that all data from across the cohort can be presented on the single average joint surface. Note that the average patch is only used as a means to display validation results from the algorithm in 3D on a common surface, and in this case is of no experimental consequence.

#### Reproducibility imaging data acquisition

40 clinical CT imaging sets were sourced from an existing control cohort of volunteer female patients in Cambridge consented for use of their imaging data in the investigation of hip disease (FEMCO: LREC 07-H0305-61; MRC-Hip fx and MRC-Ageing: LREC 06/Q0108/180; MRC-Stroke: LREC 01/245; ACCT-1: LREC 04/Q0108/257). Exclusion criteria for these studies were: dementia/cognitive impairment, unconsciousness, terminal illness, metastatic cancer, previous hip replacement (synthetic material at either hip), previous hip fracture, osteomyelitis, bone tumour, known history of metabolic bone disease, those taking oral corticosteroids, women already enrolled in a study involving x-rays. These 40 hips were selected to give a range of disease scores as measured in their digitally reconstructed radiographs^10^. The minimum joint space width range was 0 to 4.2 mm, K&L score range 0 to 3 (none to moderate). The age in this subcohort was a mean ± SD of 66 ± 17 years.

CT imaging was performed on a range of clinical CT scanners (Siemens SOMATOM Sensation 16, Siemens SOMATOM Sensation 64, Siemens SOMATOM Definition Flash, Siemens SOMATOM Definition AS, GE Medical Systems Discovery 690). This meant there were various acquisition parameters, but important reconstruction parameters were a consistent matrix of 512 × 512 covering both hips at least from superior acetabulum to below lesser trochanter, pixel spacing varying from 0.59 to 0.79 mm, slice thickness from 0.75 to 1.5 mm, and a smooth body reconstruction kernel.

Imaging data was anonymised, and either the left or right hip randomly allocated for analysis (20 of each). This heterogeneity intended to reflect a non-standardised sample in order to test reproducibility maximally between individual users of JSM. Manual proximal femur segmentation had already been performed by experienced users for a separate study investigating the effects of focal osteoporosis in the proximal femur^28^. R.H. and T.D.T. were debriefed together on the JSM technique, performed 10 test cases, then came together to review performance. Each then performed a blinded run of JSM on the same 30 hips, with results from each registered and transferred to the average surface.

#### Reproducibility metrics

Global mean reproducibility bias between users is calculated from the registered data as R.H. values minus T.D.T. values. Global 95% agreement limits are presented as 1.96 × SD of the reproducibility bias. Global RMSCV is calculated as $100\times \sqrt{({\rm{m}}{\rm{e}}{\rm{a}}{\rm{n}}\,{\rm{o}}{\rm{f}}\,{\rm{a}}{\rm{l}}{\rm{l}}\,{\rm{C}}{{\rm{V}}}^{2})}$, with individual CVs calculated as (SD/mean). Reproducibility bias, limits of agreement, and RMSCVs are also presented on the average joint space patch to show their variation in 3D around the joint space.

#### Availability of material and data

- Stradwin is freely available to download from http://mi.eng.cam.ac.uk/~rwp/stradwin/.
- wxRegsurf is freely available to download from http://mi.eng.cam.ac.uk/~ahg/wxRegSurf/.
- The MATLAB_2013a GUI written by T.D.T. is freely available on request to the corresponding author.
- All CT and HRpQCT imaging data from the technical validation experiment are anonymised but are not publicly available due to protection of patient identity and confidentiality; reasonable requests for sharing can be made with the support of the authors to the University of Cambridge Human Biology Research Ethics Committee (HBREC 2013.09), Cambridge, UK.
- All CT imaging data from the reproducibility experiment are anonymised but are not publicly available due to protection of patient identity and confidentiality; reasonable requests for sharing can be made with the support of the authors to the relevant research ethics committees: FEMCO: LREC 07-H0305-61; MRC-Hip fx and MRC-Ageing: LREC 06/Q0108/180; MRC-Stroke: LREC 01/245; ACCT-1: LREC 04/Q0108/257.
- CT imaging data used for Fig. 6a,c are anonymised but are not publicly available due to protection of patient identity and confidentiality; reasonable requests for sharing can be made with the support of the authors to the Cambridge University Hospitals NHS Foundation Trust Research and Development Department, Cambridge U.K.
- CT imaging data from Fig. 6b are fully anonymised but are not publicly available due to protection of patient identity and confidentiality; reasonable requests for sharing can be made with the support of the authors to Dr Neil Segal, University of Kansas, U.S.A.
- All acetabular joint space objects and their respective joint space measurement and measurement error data files from all experiments have been made available on line through Apollo, the University of Cambridge Open Access repository as linked to this publication (10.17863/CAM.21666).

## Electronic supplementary material

Supplementary figures
