# Supplementary material for: A new quantitative 3D approach to imaging of structural joint disease
Source: Sci Rep. 2018 Jun 18;8:9280. doi: 10.1038/s41598-018-27486-y (PMC6006324; doi:10.1038/s41598-018-27486-y)
Supplement: Supplementary file 1 — Supplementary figures [file 41598_2018_27486_MOESM1_ESM.docx]

**Title**

A new quantitative 3D approach to imaging of structural joint disease

**Authors**

T. D. Turmezei^a,b^*, G. M. Treece^a^, A. H. Gee^a^, R. Houlden^c^, K. E. S. Poole^c^.

**Affiliations**

^a^Cambridge University Engineering Department, Cambridge, UK.

^b^Department of Radiology, Norfolk and Norwich University Hospital, Norwich, UK.

^c^Department of Medicine, University of Cambridge, Cambridge, UK.

*tom.turmezei@nnuh.nhs.uk

**Supplementary Figures**

**
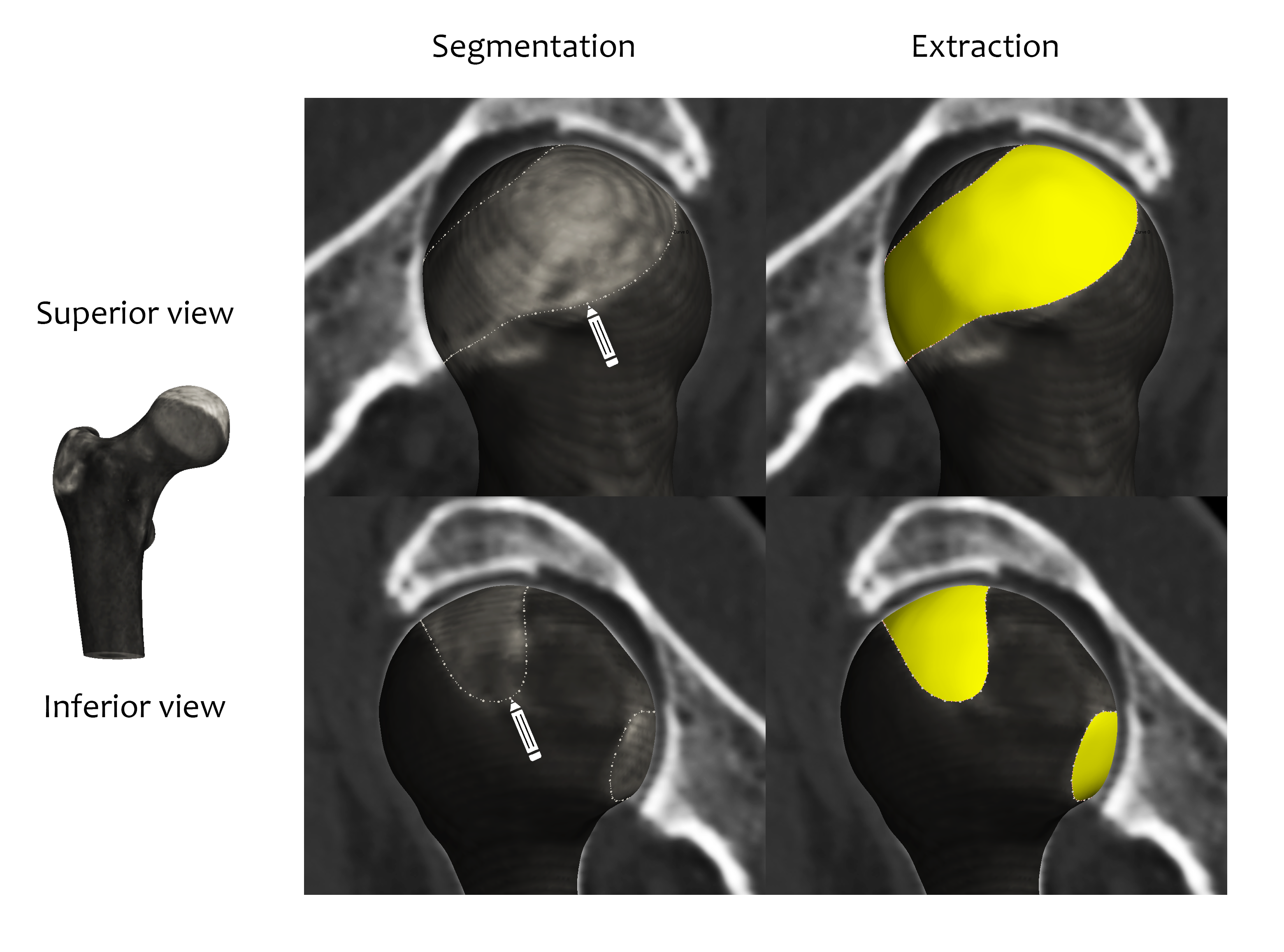
**

**Fig. S1 Magnified view of joint space boundary segmentation.** Magnified views of step 5 from Fig. 1, showing superior (top) and inferior (bottom) views of the hip joint, with the proximal femoral surface object displaying bright patches where there is overlying acetabular bone, as described in step 4. Segmentation of the joint space boundary (left) is performed in Stradwin using a 3D surface segmentation tool guided by freehand, user-manipulated MPR (background imaging data in the figure) to outline the patch. This MPR plane can be rotated around the femoral head to help track the boundary of the joint as it is segmented. The 3D “joint space patch” (yellow) is then extracted from the proximal femoral surface (right), as described in step 6.


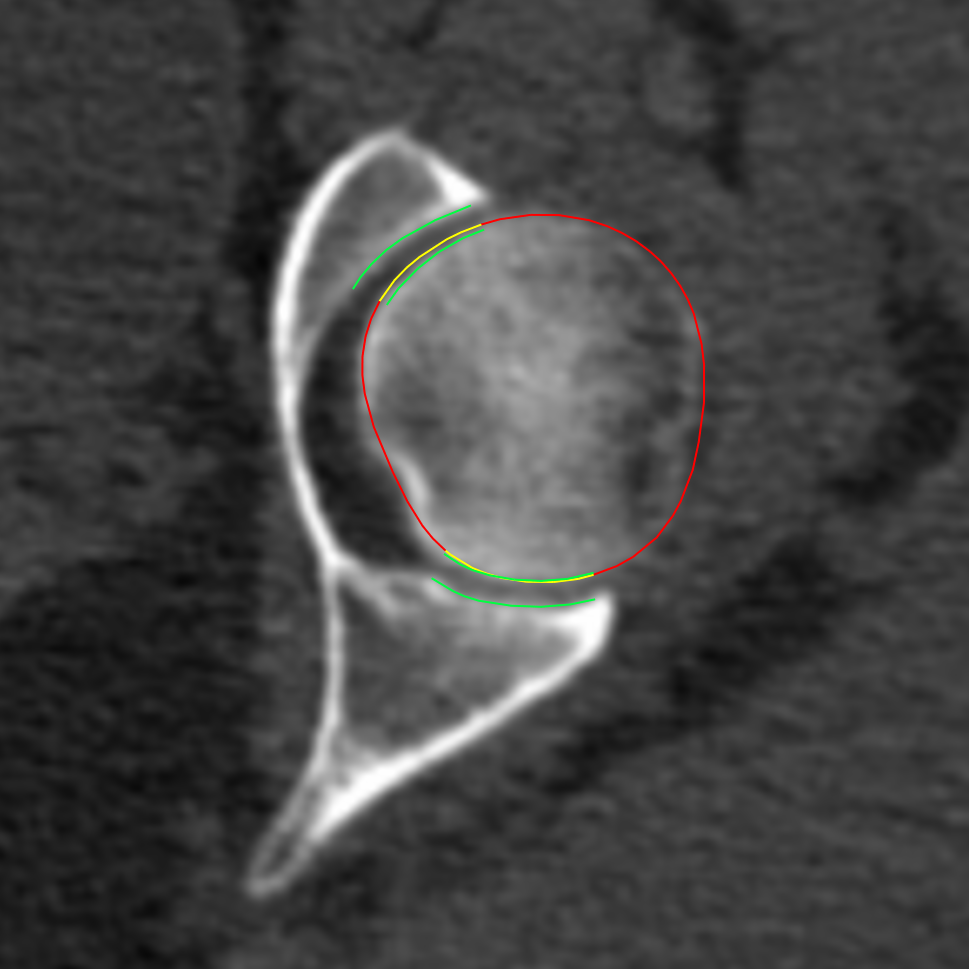


**Fig. S2 Comparison of the joint space patch and output surfaces.** Axial plane view of the left hip from the same patient as in Fig. 5a (no radiological hip disease). This shows the in-plane outline of the original femoral surface (red), the joint space patch cut from this (yellow) and the JSM algorithm output joint surfaces (green). Note how the joint space patch is not the same as the output femoral or acetabular joint surfaces, and is only used as the basis to deliver these using the JSM algorithm. However, the patch is important for restricting the measurement points within the manually segmented joint space edge in step 5, shown as a magnified view in Fig S1.

**
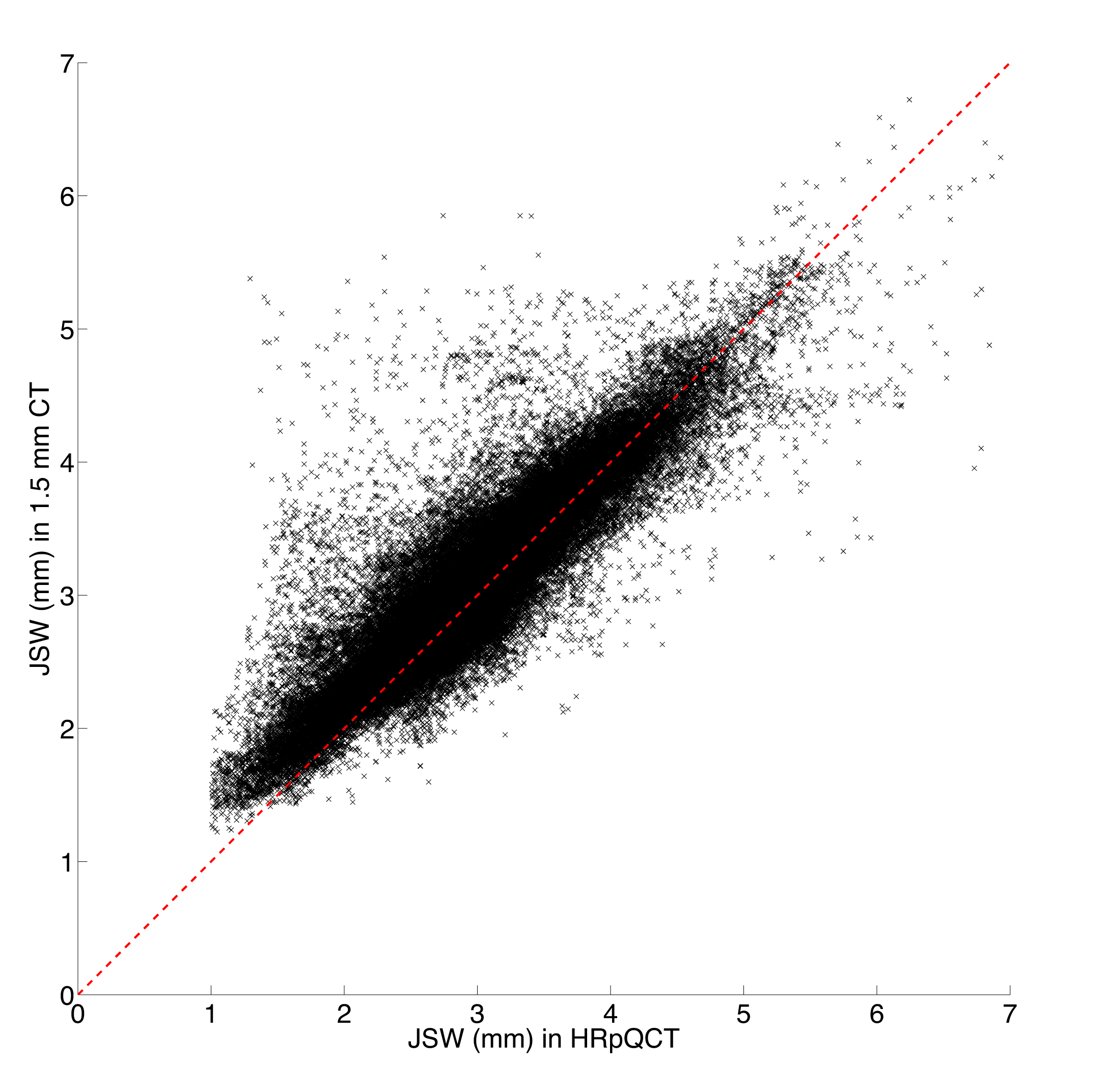
**

**Fig. S3 Scatter plot for technical validation data.** Clinical CT *vs* HRpQCT validation data presented as a scatter plot, corresponding to Fig 3a but without upper and lower 2.5% data limits. The red dashed line of y = x is the target truth for validation (i.e. a value measured in 1.5 mm CT equals that measured in HRpQCT).
